# Single-cell selectivity and functional architecture of human lateral occipital complex

**DOI:** 10.1371/journal.pbio.3000280

**Published:** 2019-09-12

**Authors:** Thomas Decramer, Elsie Premereur, Mats Uytterhoeven, Wim Van Paesschen, Johannes van Loon, Peter Janssen, Tom Theys

**Affiliations:** 1 Laboratory for Neuro- and Psychophysiology, KU Leuven and the Leuven Brain Institute, Leuven, Belgium; 2 Department of Neurosurgery, University Hospitals Leuven, Leuven, Belgium; 3 Research Group Experimental Neurosurgery and Neuroanatomy, KU Leuven and the Leuven Brain Institute, Leuven, Belgium; 4 Department of Neurology, University Hospitals Leuven, Leuven, Belgium; 5 Laboratory for Epilepsy Research, KU Leuven, Leuven, Belgium; McGill University, CANADA

## Abstract

The human lateral occipital complex (LOC) is more strongly activated by images of objects compared to scrambled controls, but detailed information at the neuronal level is currently lacking. We recorded with microelectrode arrays in the LOC of 2 patients and obtained highly selective single-unit, multi-unit, and high-gamma responses to images of objects. Contrary to predictions derived from functional imaging studies, all neuronal properties indicated that the posterior subsector of LOC we recorded from occupies an unexpectedly high position in the hierarchy of visual areas. Notably, the response latencies of LOC neurons were long, the shape selectivity was spatially clustered, LOC receptive fields (RFs) were large and bilateral, and a number of LOC neurons exhibited three-dimensional (3D)-structure selectivity (a preference for convex or concave stimuli), which are all properties typical of end-stage ventral stream areas. Thus, our results challenge prevailing ideas about the position of the more posterior subsector of LOC in the hierarchy of visual areas.

## Abstract 2

Microelectrode recordings in the human lateral occipital complex (LOC) yield highly selective single-unit, multi-unit and high-gamma responses to images of objects; contrary to predictions from functional imaging studies, all neuronal properties indicated that this posterior subsector of LOC occupies an unexpectedly high position in the hierarchy of visual areas.

## Introduction

Our understanding of the human brain is hampered by the limitations imposed upon neuroscience research in humans. Noninvasive measurements of brain activity (Electroencephalography [EEG], functional magnetic resonance imaging [fMRI]) often provide only coarse information regarding neural activity, due to their limited spatial or temporal resolution. Genuine insight into the function of a brain area requires detailed measurements of the electrical activity of individual neurons and small populations of neurons at high spatiotemporal resolution. Intracortical electrophysiological recordings in humans are scarce, therefore the human visual cortex is virtually unexplored at the level of the individual neurons and small populations of neurons. Several studies have recorded field potentials with intracranial electrodes [1–3], but macroelectrode recordings still reflect activity of hundreds of thousands of neurons and—due to their large contact area—cannot measure spiking activity, nor can they reveal the microarchitecture of visual cortex on a submillimeter scale. A series of studies using depth electrodes in the mesial temporal lobe have investigated the visual responses of single neurons in entorhinal and perirhinal cortex [4–9], and one study [10] reported single-unit responses during imagined actions (reaching and grasping) in a patient with a microelectrode array implanted in parietal cortex, who thereby obtained accurate control over a robot arm. Self and colleagues [11] measured multi-unit activity (MUA) and local field potential (LFP) activity in early visual areas (V2/V3) in a patient using hybrid macro-micro depth electrodes. This study observed that the properties of populations of neurons (multi-unit receptive fields [RFs], tuning for contrast, orientation, spatial frequency, and modulation by context and attention) were similar to those of neurons in the macaque areas V2 and V3. To our knowledge, intracortical recordings in intermediate human visual areas such as the lateral occipital complex (LOC) have never been performed.

Recordings in patients, combined with similar measurements in monkeys, may allow us to answer very specific questions with regard to the properties of individual neurons and the homologies between cortical areas in humans and monkeys. For example, assessing the shape selectivity or the RF profile of LOC neurons requires intracortical recordings, which have never been performed. Moreover, despite 2 decades of functional imaging studies in both species [12], the homologies between the (subsectors of) LOC and ventral occipitotemporal cortex in humans and the monkey inferior temporal cortex (ITC) areas have not yet been resolved. The LO1 and LO2 subsector may be retinotopically organized [13,14], similar to the posterior part of macaque inferotemporal cortex (TEO) [15], but direct single-cell evidence in the two species is lacking. Furthermore, the microarchitecture of these areas in humans (i.e., the spatial clustering of shape selectivity on the scale of cortical columns measuring 0.5 mm) is very difficult to assess with fMRI [16]. To investigate the clustering of neuronal selectivity in human visual cortex, recordings with intracortical microelectrodes are necessary.

## Results

**Fig 1:**
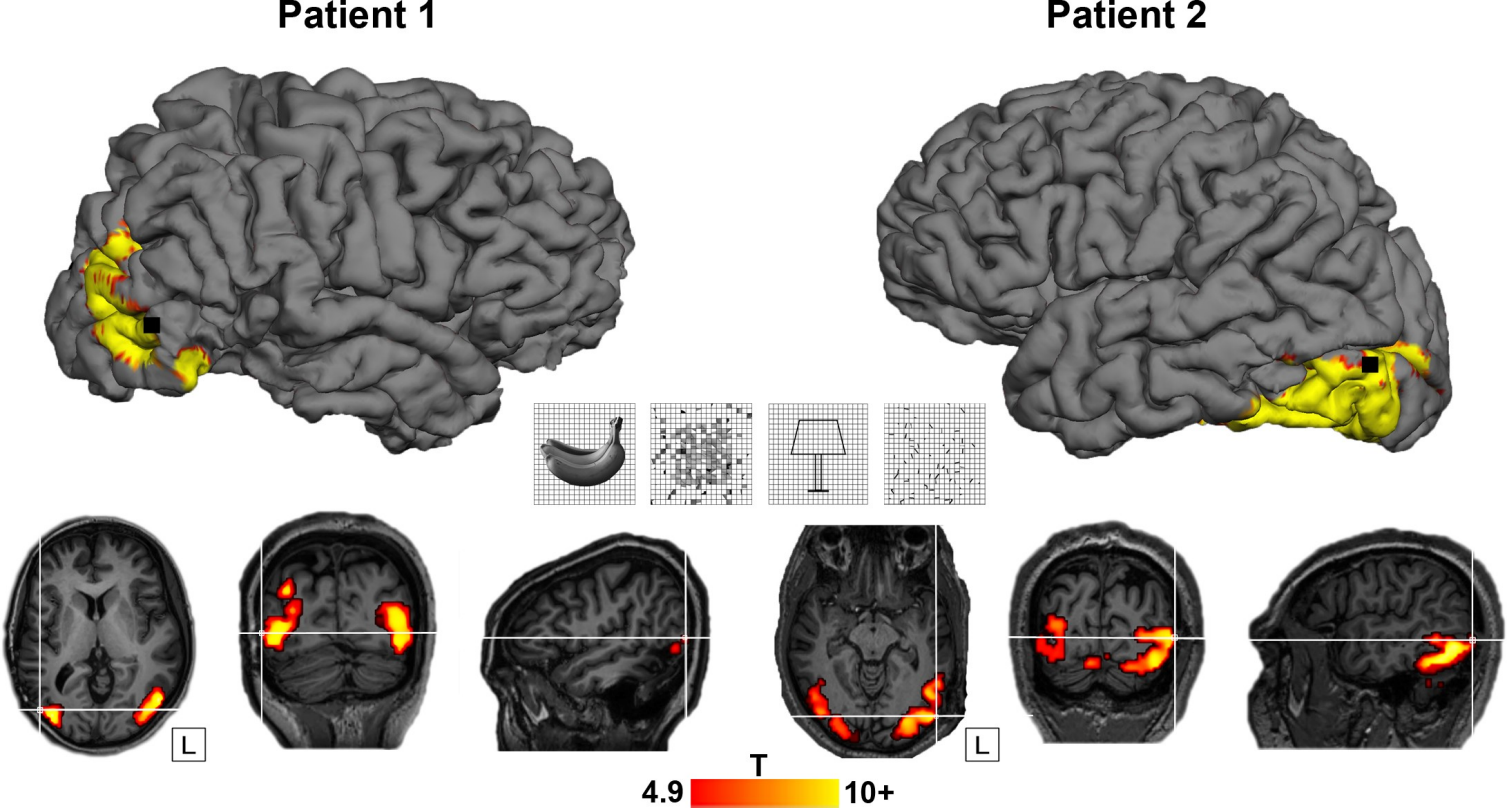
LOC localizer and location of arrays. Top: site of Utah array implantation (black) projected onto 3D rendering of the brain in patient 1 (left) and patient 2 (right), overlayed with the fMRI LOC localizer activation (contrast [shapes + outlines] − [scrambled shapes + scrambled outlines]; *p* < 0.05, FWE-corrected for multiple comparisons). Center: LOC localizer stimuli (shapes, scrambled shapes, outlines, scrambled outlines), see [17]. Bottom: axial, coronal, and sagittal view. T-values plotted on T1-weighted image. Crosshair indicates the position of the Utah array in relation to the fMRI activation for both patients. The underlying data can be found at doi:10.5061/dryad.qd4vd71. 3D, three-dimensional; fMRI, functional magnetic resonance imaging; FWE, family-wise error; LOC, lateral occipital complex.

In 2 patients who were evaluated for refractory epilepsy, we ran an LOC-localizer fMRI experiment, in which blocks of nonscrambled shapes and outlines were interleaved with control blocks of scrambled stimuli (Fig 1 center [17]). A 96-channel Utah microelectrode array was implanted in the LOC (Fig 1A; Montreal Neurological Institute [MNI] coordinates 55, −71, 1 for patient 1 and −55, −77, 6 for patient 2) [14]. Table 1 illustrates the position of the array in both patients in relation to previous studies. We verified the anatomical location of the array using a computed tomography (CT) scan obtained after array implantation, which was coregistered onto the anatomical MRI. Fig 1B shows the fMRI activations in the 2 patients for the contrast nonscrambled versus scrambled stimuli, plotted on the patient's own anatomical MRI (*p* < 0.05, family-wise error [FWE] corrected). The fMRI results confirmed that the microelectrode arrays were indeed implanted in the hotspot of fMRI activations.

**Table 1:** MNI coordinates. Position of the micro-electrode array in relation to the centroid of fMRI activations in the literature [18,19]. Coordinates are shown in MNI space for the LH and RH.

	LH			RH		
	x	y	z	x	y	z
Patient 1				55	−71	1
Patient 2	−55	−77	6			
LOC ant	−46	−54	−11	29	−49	−16
LOC post	−59	−82	6	31	−82	−9
LO1	−31	−93	−4	31	−92	−3
LO2	−38	−85	−6	37	−84	−6
hMT	54	−74	14	21	−67	3

**Abbreviations:** ant, anterior; fMRI, functional magnetic resonance imaging; hMT, human middle temporal visual area; LH, left hemisphere; LO1, subsector 1 of lateral occipital complex; LO2, subsector 2 of lateral occipital complex; LOC, lateral occipital complex; MNI, Montreal Neurological Institute; post, posterior; RH, right hemisphere

### Effect of image scrambling

**Fig 2:**
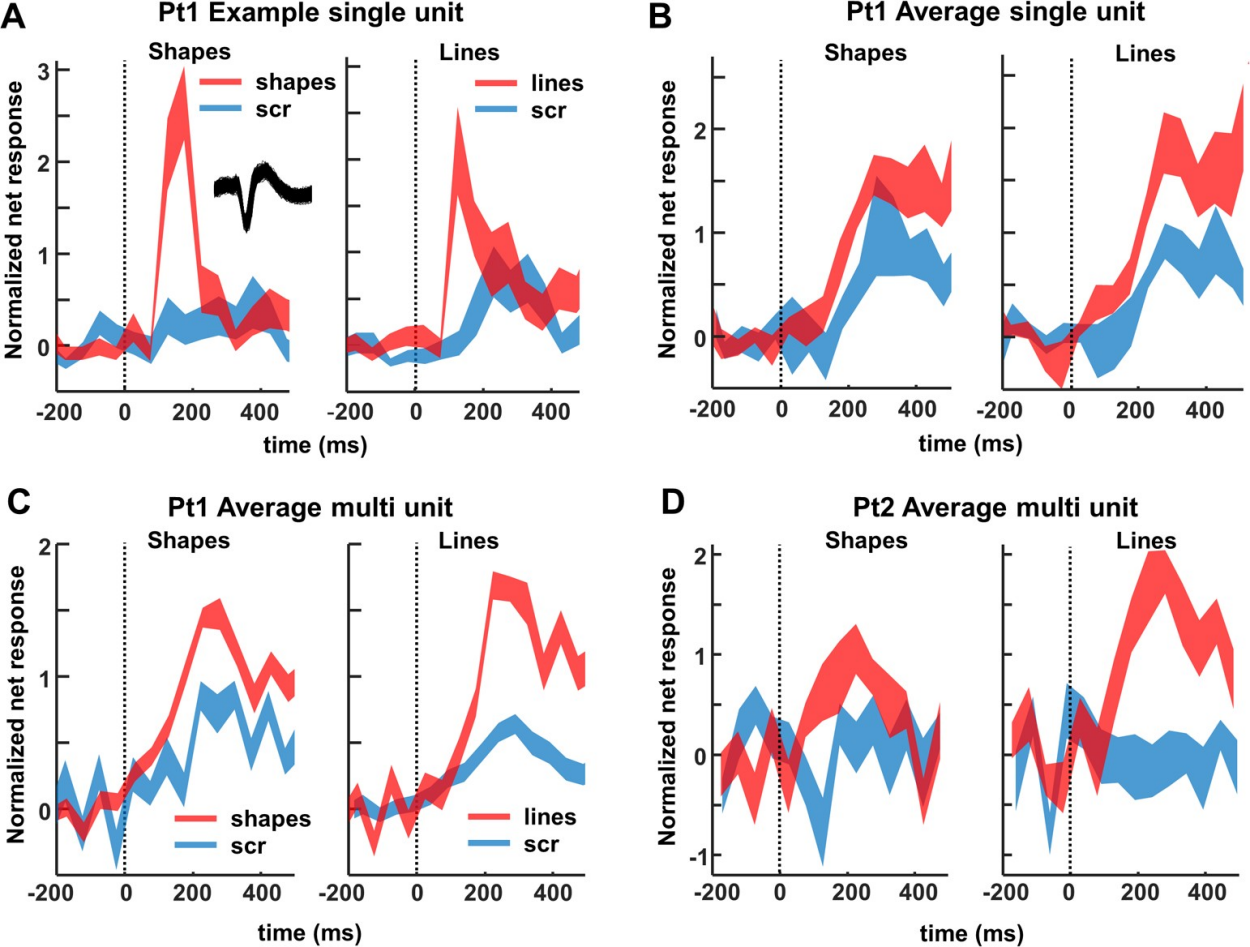
Single- and multi-unit normalized net response. Net spike rate divided by the average spike rate for the best condition (50–300 ms after stimulus onset) to images of objects and scrambled controls. (A) Example neuron. Average response to intact (red) and scrambled (blue) shapes (left) and line stimuli (right). The inset illustrates the spike waveform. (B) Average single-unit responses across all visually responsive channels in patient 1. (C, D) Average multi-unit responses to intact and scrambled stimuli in patient 1 and 2, respectively, across all visually responsive channels. The underlying data can be found at doi:10.5061/dryad.qd4vd71. Pt1, patient 1; Pt2, patient 2; scr, scrambled.

The example neuron in Fig 2A responded significantly more strongly to images of objects (both shapes and outlines) compared to scrambled controls (permutation test: *p* < 0.001, d′ = 0.72 for shapes and *p* = 0.002, d′ = 0.50 for line stimuli). Unlike previous recordings in the human medial temporal lobe [5,8], this neuronal response was brisk and relatively transient (response and selectivity latency: 125 ms). The large waveform (inset in Fig 2A) indicates that this neuron was well isolated.

We recorded neuronal responses to images of objects in patient 1 in 2 separate sessions (number of channels: 174) and detected 42 visually responsive single units (average normalized net response in Fig 2B). Entirely consistent with the fMRI results, half of these neurons (21 of 42) responded significantly more strongly to intact than to scrambled images of objects (permutation test nonscrambled versus scrambled: *p* < 0.05; median d′ index: 0.35), and no single unit showed a significant preference for scrambled controls. The median response latency of these 21 selective neurons (calculated on the responses to intact shapes and outlines) was 150 ms, whereas the fastest neurons (percentile 10) started to respond at 75 ms after stimulus onset. However, the median selectivity latency (i.e., the first bin with significant response differences between intact and scrambled images) was much higher (225 ms), and no neuron started to discriminate between intact and scrambled stimuli before 75 ms. We obtained highly similar results for MUA in both patients (Fig 2C and 2D). A large number (46 of 83) of the visually responsive channels preferred intact over scrambled shapes (41 of 71 for patient 1; 5 of 12 for patient 2; permutation test: *p* < 0.05; median d′: patient 1: 0.45, patient 2: 0.28). Not surprisingly, the average multi-unit response of all visually responsive channels was greater for nonscrambled stimuli than for scrambled controls (*p* < 0.001, permutation test, Fig 2C and 2D).

**Fig 3:**
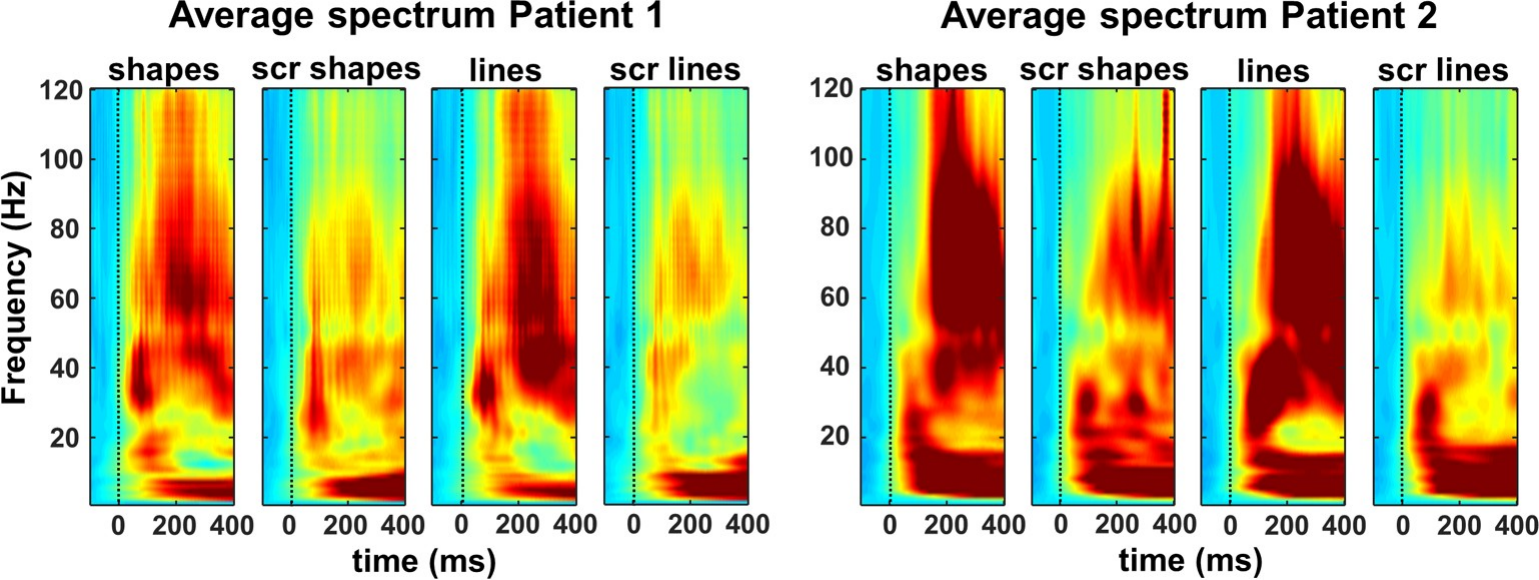
Time-frequency plots of the average LFPs. Indicating that the high-gamma (80–120 Hz) power is significantly stronger for intact shapes and outlines than for their scrambled controls. The underlying data can be found at doi:10.5061/dryad.qd4vd71. LFP, local field potential; scr, scrambled.

When looking at the LFP signal that was recorded together with the spiking activity, virtually all channels responded significantly to visual stimulation (80–120 Hz, or high-gamma power, intact shapes versus prestimulus baseline; permutation test, *p* < 0.05; patient 1: *n* = 164/174 or 94%; patient 2: *n* = 269/285 or 94%). On average, we observed a broad-band response after stimulus onset in all 4 conditions (permutation test, *p <* 0.01, corrected for multiple comparisons, Fig 3), in which the LFP response to intact shapes and outlines was significantly stronger than to scrambled controls, both at the level of the average high-gamma power and on the great majority of the individual channels (permutation test, *p* < 0.001; patient 1: 109 /174 [63%] individual channels; patient 2: 242/285 [85%] individual channels). As expected, the lower frequency bands discriminated less reliably between intact and scrambled shapes. Thus, single-unit activity (SUA), MUA, and LFP data clearly demonstrate that neurons in human LOC are more responsive to intact shapes than to scrambled shapes, confirming and validating the results of the fMRI localizer.

### Shape selectivity and spatial clustering

**Fig 4:**
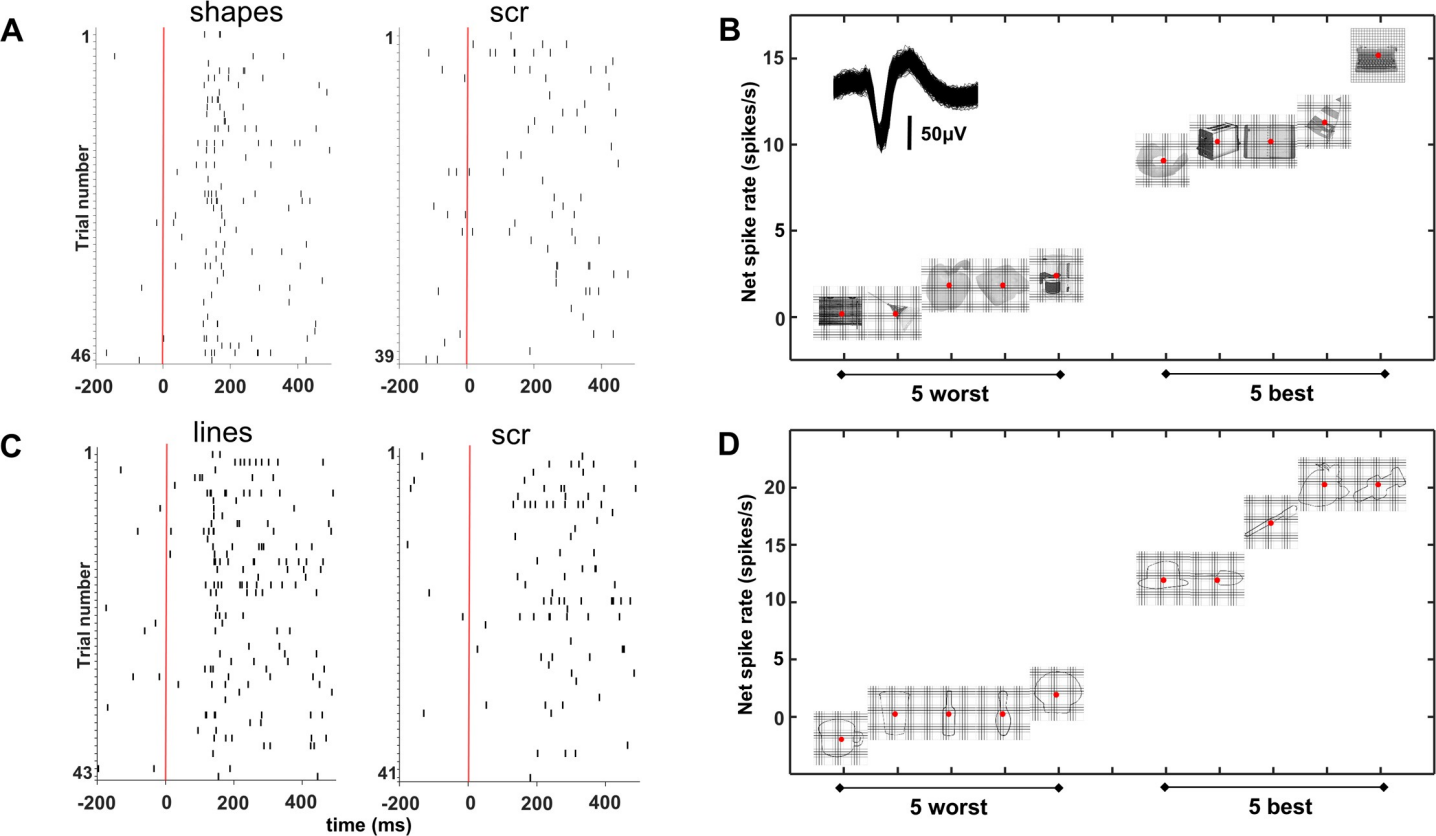
Example neuron. (A, C) This neuron responded more to intact shapes and lines than to their scrambled versions as shown in these spike raster plots. (B, D) Response to the 5 best and 5 worst stimuli, indicating that this neuron discriminated reliably between different shapes. scr, scrambled.

**Fig 5:**
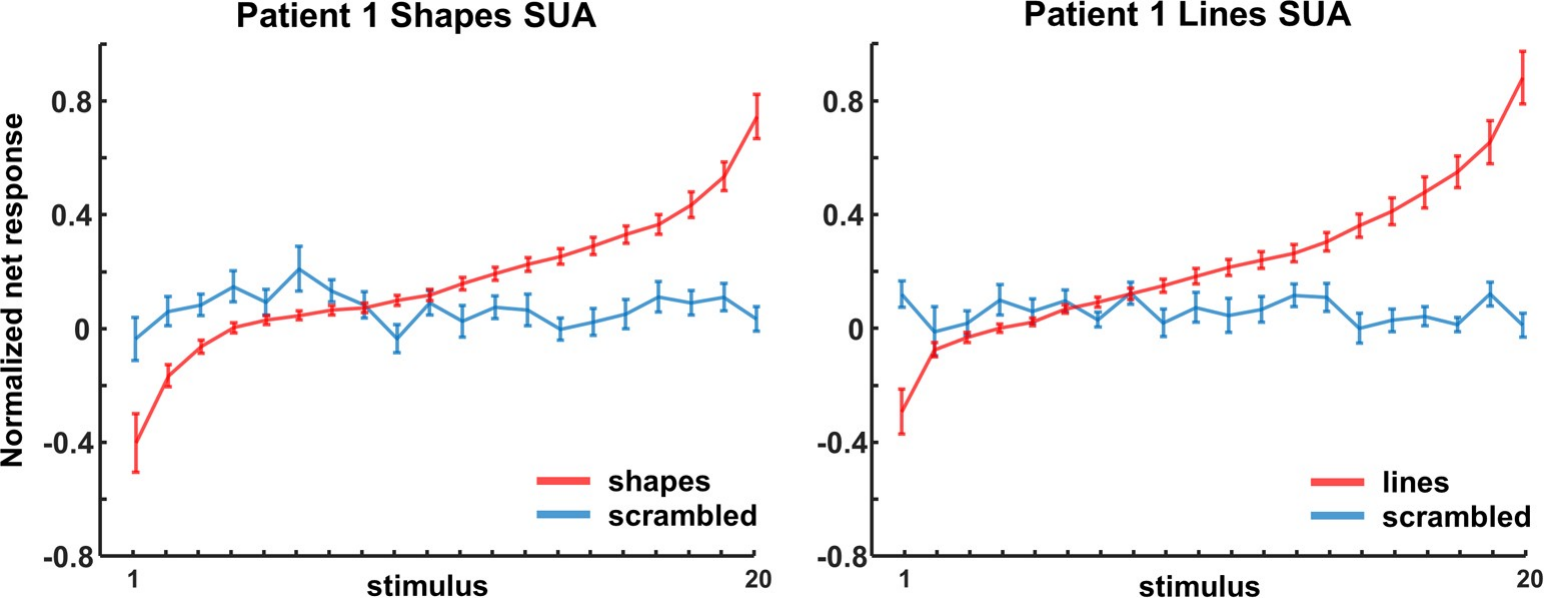
Ranking SUA. Ranking analyses of shapes and lines for SUA in patient 1. The same ranking is applied for the corresponding scrambled control stimuli. The underlying data can be found at doi:10.5061/dryad.qd4vd71. SUA, single-unit activity.

**Fig 6:**
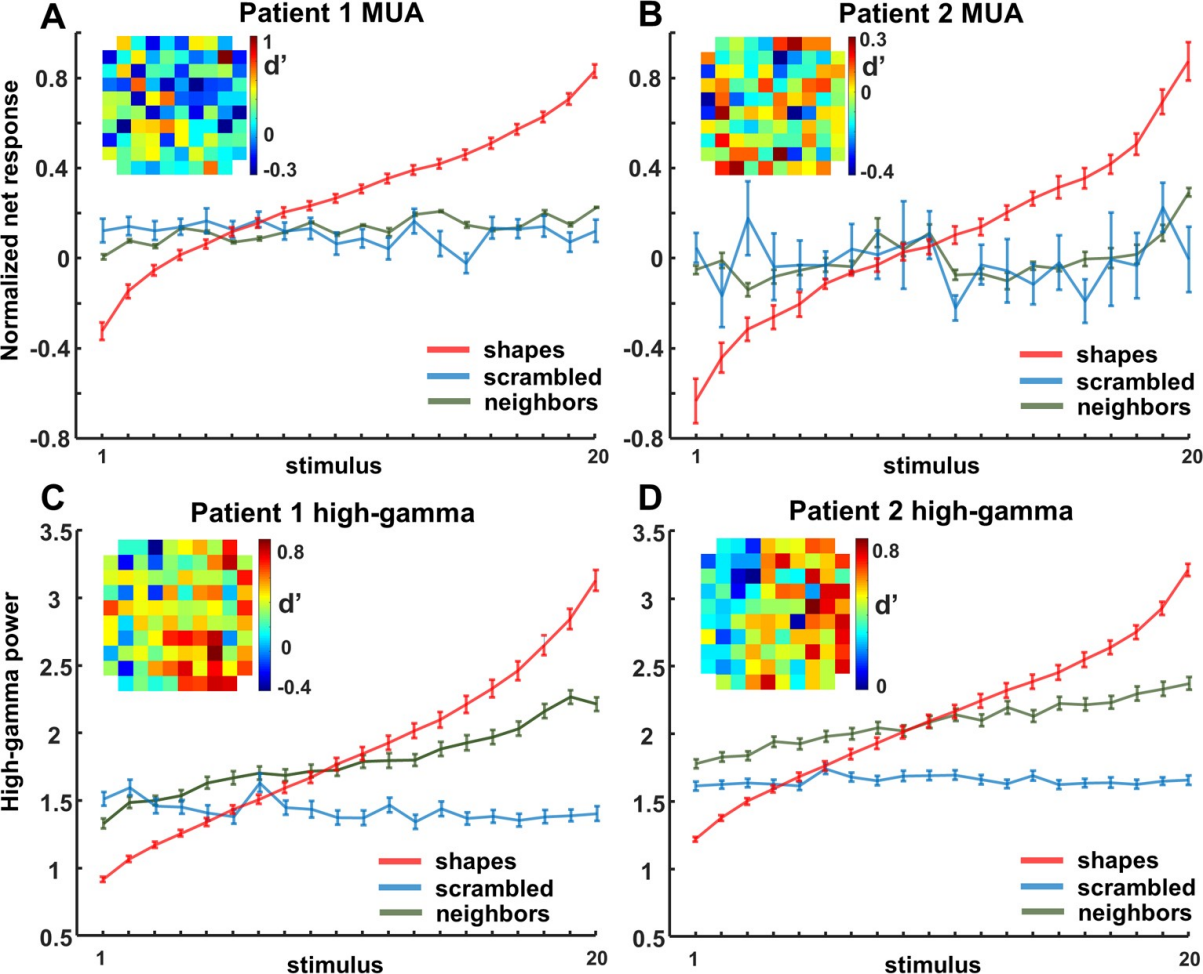
Ranking MUA/high gamma. Ranking of shapes for MUA and high-gamma LFP for each patient. The same ranking is applied for the neighboring channels and the corresponding scrambled control stimuli. An overview of the array is shown as inset; each square represents an electrode, illustrating that electrodes with high d′ values (response nonscrambled versus scrambled) are neighbored by electrodes with low d′ values. The underlying data can be found at doi:10.5061/dryad.qd4vd71. LFP, local field potential; MUA, multi-unit activity.

Both SUA (2/21) and MUA (8/46) recording sites were not only sensitive to image scrambling but could also be selective for individual shapes (one-way ANOVA with factor stimulus number *p* < 0.05). The example neuron in Fig 4 not only responded more to intact shapes and lines than to their scrambled versions (Fig 4A and 4C) but also discriminated reliably between different shapes (Fig 4B, inset shows waveform of the neuron) and line stimuli (Fig 4D). In this neuron, the 5 most effective stimuli elicited responses between 10 and 20 spikes/s, whereas the least effective stimuli evoked virtually no response (see S1 Fig for a second example neuron.) To quantify and visualize this shape selectivity, we ranked the intact shapes based on the SUA responses (for all 21 channels with significant selectivity for image scrambling) and calculated the average SUA response to the ranked intact shapes and to the corresponding scrambled images (Fig 5A and 5B). Despite the fact that we did not search for selective neurons, shape selectivity was nonetheless robust, since the half-maximum response was measured for rank 17 (shapes) and 16 (lines), and the least-preferred shapes even evoked inhibitory responses. No significant tuning was present for the corresponding scrambled controls (see Table 2 for linear regression slopes). The results were highly similar for the MUA responses in both patients (*N* = 46, Fig 6A and 6B and S2 Fig).

**Table 2:** Slope of regression lines with 95% confidence interval for shapes and lines. The same ranking was applied for the scrambled versions of the stimuli and for their neighboring channels, respectively.

Condition	Patient 1			Patient 2	
	*SUA*	*MUA*	*LFP*	*MUA*	*LFP*
**Shapes**	0.0404 (0.0337 to 0.047)	0.049 (0.0448 to 0.0531)	0.1015 (0.0932 to 0.1097)	0.0623 (0.0561 to 0.0685)	0.0892 (0.0834 to 0.095)
**Applied ranking for scr_shapes**	−0.0008* (−0.0058 to 0.0042)	−0.0026* (−0.0064 to 0.0011)	−0.0077* (−0.0129 to −0.0026)	−0.0005* (−0.0098 to 0.0087)	0.0002* (−0.0027 to 0.0031)
**Applied ranking for neighbors**	N/A	0.0072* (0.0043–0.0101)	0.041* (0.0362–0.0458)	0.0082* (0.001–0.0153)	0.0285* (0.026–0.0309)
**Lines**	0.0443 (0.038 to 0.0507)	0.0468 (0.0431 to 0.0505)	0.0974 (0.0867 to 0.108)	0.0469 (0.0432 to 0.0506)	0.0919 (0.082 to 0.1018)
**Applied ranking for scr_lines**	−0.0009* (−0.289 to −0.0047)	0.1483* (−0.0028 to −0.0056)	−0.0059* (−0.0094 to −0.0024)	0.0073* (−0.0047 to 0.0194)	−0.0005* (−0.0043 to 0.0034)
**Applied ranking for neighbors**	N/A	−0.0006* (−0.0057 to 0.0045)	0.0312* (0.0279 to 0.0346)	0.0007* (−0.0116 to 0.0256)	0.0305* (0.0262 to 0.0347)

*Indicates significant ranking difference.

**Abbreviations:** LFP, local field potential; MUA, multi-unit activity; N/A, not applicable; scr, scrambled; SUA, single-unit activity

The high-gamma responses to intact shapes were equally selective (Fig 6C and 6D; one-way ANOVA with factor stimulus number, for shapes: *p* < 0.05 in 15/109 for patient 1; 72/242 channels for patient 2). The most effective intact shape elicited 3 to 4 times more high-gamma activity than the least-preferred shape (half-maximum response for rank 15 in both patients), and no significant tuning was present for the corresponding scrambled controls.

Many MUA and LFP sites were sensitive to image scrambling, but the degree of sensitivity differed markedly on neighboring channels spaced a mere 400 microns apart. This unexpected spatial specificity in the MUA and high-gamma responses to image scrambling became evident in the d′ indices mapped on the spatial layout of the array (compare, e.g., the d′ of a highly selective channel to its neighboring channels, Fig 6A–6D, insets). To quantify this spatial specificity to image scrambling across the array, we calculated a two-way ANOVA for the evoked high-gamma responses for each of the 109 (patient 1) or 240 (patient 2) selective channels with factors scrambling (scrambled versus non-scrambled) and position (neighboring position). In the large majority of the channels, the main effect of position (patient 1: *N* = 108/109; patient 2: N = 235/240) or the interaction between the factors scrambling and position (patient 1: N = 57/109; patient 2: N = 205/240) were significant (p < 0.05, highly similar results were found for MUA).

We also observed a high degree of spatial clustering for shape preference across the array, both at the level of MUA and at the level of high-gamma responses. For each responsive channel (the center electrode), we calculated the average responses to the intact shapes on all its neighboring channels based on the shape ranking of the center electrode (Fig 6, green lines). The shape preference differed markedly between each center electrode and its neighbors (Fig 6A–6D, see Table 2), indicating that the shape preference of human LOC neurons is clustered on a submillimeter scale, similar to the monkey ITC [20,21].

### Receptive fields

**Fig 7:**
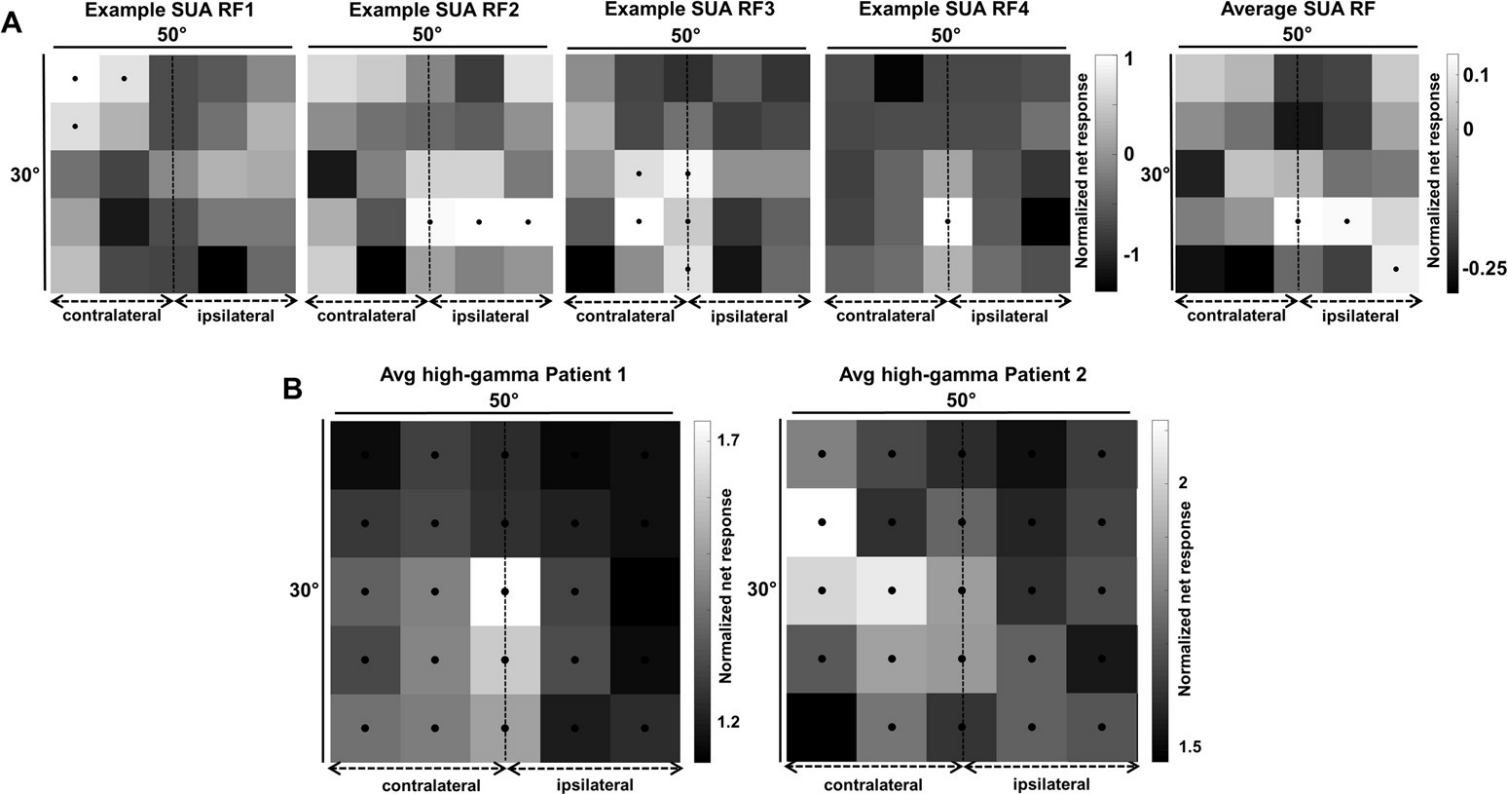
RF maps. (A) Single-unit data. The first 4 panels show the RFs of 4 example neurons. All responses are normalized to the maximum visual response. Black dots indicate responses higher than 50% of the maximum response (per channel). Rightmost panel: average RF for all visually responsive channels. (B) Average RF for high-gamma responses for patient 1 (left) and patient 2 (right). The underlying data can be found at doi:10.5061/dryad.qd4vd71. RF, receptive field; SUA, single-unit activity.

A fundamental characteristic of visual neurons is their RF. To map the RF of neurons in human visual cortex, we presented an intact shape at 25 positions on the screen covering a 30 by 50 degree area of the visual field. The 4 example neurons recorded in patient 1 (Fig 7A) clearly demonstrate that the RFs were relatively large (average surface area 473 degree^2^) and covered both the ipsi- and the contralateral hemifield. Out of 46 visually responsive single neurons (stimulus versus baseline, *p* < 0.05, permutation test), 24 (52%) responded maximally in the contralateral hemifield, 13 (28%) in the ipsilateral hemifield, and 9 neurons responded maximally at the midline (3 of which were at the fovea). Six neurons showed bilateral responses (i.e., >50% of the maximal response). The average RF (rightmost panel in Fig 7A) at the single-neuron level included the fovea and the ipsilateral hemifield. The average RF profile was similar when determined using the high-gamma responses (Fig 7B): in both patients, the high-gamma RF contained the center of the visual field, and visual responses (at >50% of the maximum response) were present both contra- and ipsilaterally. Thus, the average RF in this part of the human LOC was consistently large and bilateral.

### Three-dimensional structure selectivity

**Fig 8:**
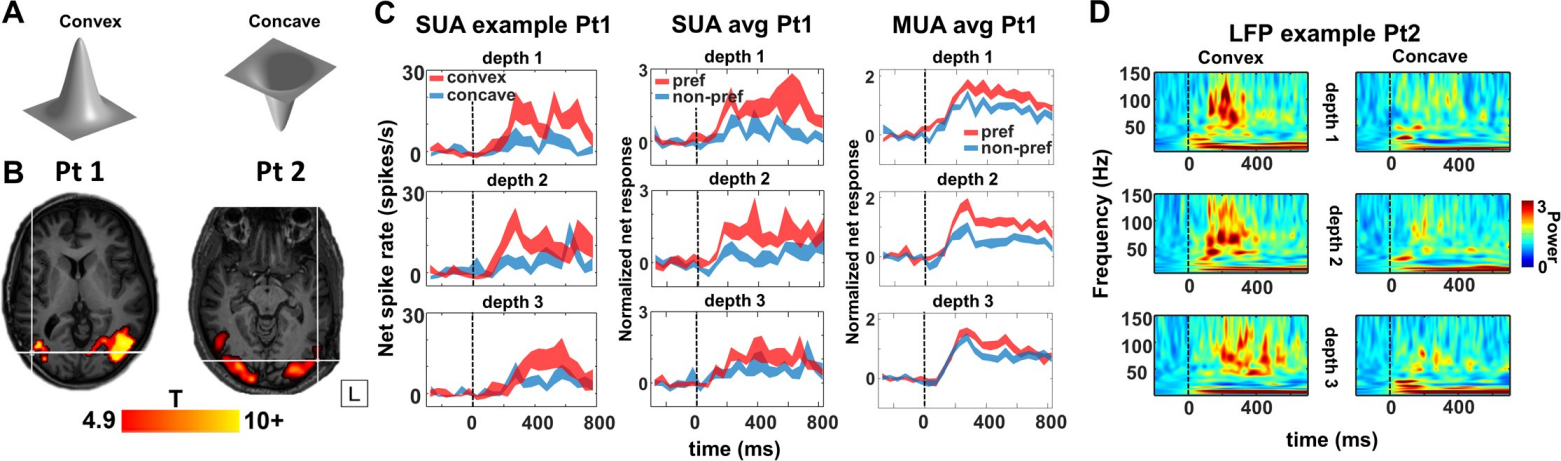
Stereo experiment. (A) Stimuli. (B) T-values for main effects of stereo, contrast [curved stereo + flat stereo]–[curved control + flat control], plotted on T1-weighted image. *p* < 0.05, FWE-corrected for multiple comparisons. Crosshair indicates the position of the Utah array. The overlap of the LOC localizer and stereo fMRI is shown in S4 Fig. (C) Example single neuron (left column), average single-unit responses (middle column), and average multi-unit responses (right column) to preferred (red) and nonpreferred (blue) curved surfaces at 3 positions in depth (upper row: near; middle row: center; and bottom row: far). (D) Time-frequency power spectra of an example channel in patient 2 for convex and concave stimulus presentations at 3 positions in depth. This site is selective for convex shapes across different positions in depth. The underlying data can be found at doi:10.5061/dryad.qd4vd71. fMRI, functional magnetic resonance imaging; FWE, family-wise error; LFP, local field potential; LOC, lateral occipital complex; MUA, multi-unit activity; nonpref, nonpreferred stimulus; Pt1, patient 1; Pt2, patient 2; pref, preferred stimulus; SUA, single-unit activity.

The previous results have addressed only neural responses to two-dimensional (2D) shapes, but neurons in the macaque ITC are also selective for three-dimensional (3D) stimuli [22,23], and several human fMRI studies have suggested that the LOC is sensitive to binocular disparity [24–26]. To investigate the selectivity of LOC neurons for stereo stimuli, we ran a stereo-localizer fMRI experiment, in which blocks of stereo stimuli (curved and flat surfaces at different disparities) alternated with blocks of control stimuli (the monocular images presented without disparity [27,28]). Fig 8B shows the T-values for the contrast [stereo] − [control], plotted on the anatomical MRI and CT scans of both patients with the array inserted (*p* < 0.05, FWE corrected). The fMRI results demonstrate that the microelectrode arrays, indicated by the white crosshair, were indeed implanted close to the hotspot of the disparity-related fMRI activations in the LOC.

We recorded neural activity in LOC during the presentation of stereo stimuli (2 recording sessions) while the patients were categorizing concave and convex surfaces (Fig 8A) at different positions in depth (SUA on 52 channels in patient 1). It is important to note that we analyzed the selectivity for 3D structure from stereo for stimuli with the same circumference shape, as in previous studies [22,29,30]. The example neuron in Fig 8C (left panel) preferred convex over concave shapes at all 3 positions in depth (average d′ = 0.73), indicating 3D structure (i.e., higher-order disparity) selectivity. Notice that this neuron did not start to respond until 100 ms after stimulus onset and reached its peak activity only after 250 ms. In total, we recorded 39 visually responsive single neurons in this test, 16 (41%) of which showed 3D-structure selectivity (i.e., a main effect of stereo and/or a significant interaction between stereo and position in depth with no reversal in selectivity [31]). For these 16 selective neurons (8 preferring convex), we plotted the average net responses to the preferred and nonpreferred 3D surfaces at each position in depth (Fig 8C middle panel). This population of LOC neurons preserved its selectivity at every position in depth, as did the MUA (*N =* 21 sites, right panel in Fig 8C; 14 [67%] selective for convex). Similar to the example neuron in Fig 8C, the population (SUA and MUA combined) response latency (125 ms) and the latency of the 3D-structure selectivity (275 ms) were relatively long compared to previously reported data obtained in the monkey ITC [22,29]. Moreover, both patients showed significant 3D-structure–selective high-gamma responses (15% of visually responsive channels in patient 1, 20% in patient 2, example channel in Fig 8D, average high-gamma selectivity of 3D-structure–selective sites is shown for both patients in S3 Fig; in patient 1, 8 sites [50%] preferred convex, in patient 2, 27 sites [79%] preferred convex). There was no difference in behavioral performance (percent correct), and the reaction times were similar for convex and concave shapes in both patients (one-way ANOVA with factor condition: F[5] = 1.07, *p* = 0.38).

**Fig 9:**
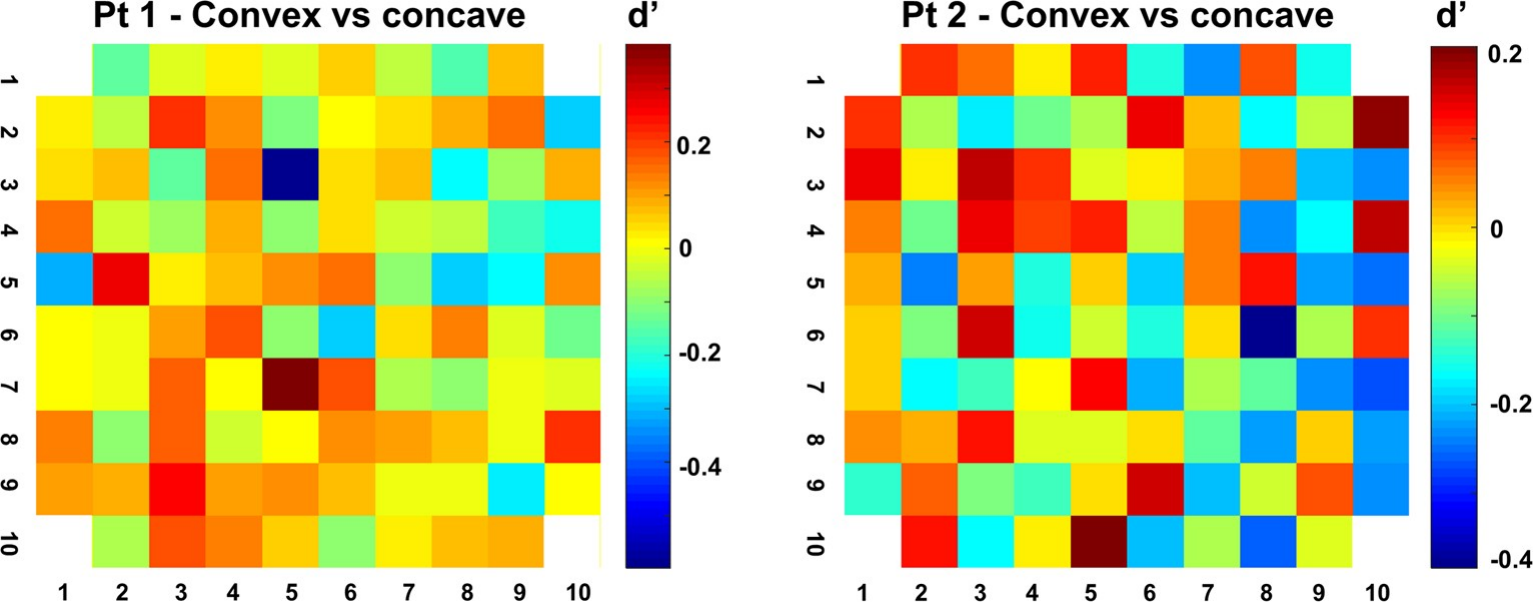
Clustering of the 3D-structure preference. d′ values (convex versus concave) of high-gamma responses across both arrays. High-gamma 3D-structure preference was highly localized on individual electrodes, since recording sites with a high d′ (convex versus concave) were frequently located next to recording sites with a low d′. The underlying data can be found at doi:10.5061/dryad.qd4vd71. 3D, three-dimensional; Pt1, patient 1; Pt2, patient 2.

Not unlike the selectivity for image scrambling and for individual shapes, the high-gamma 3D-structure preference was highly localized on individual electrodes, since recording sites with a high d′ (convex versus concave) were frequently located next to recording sites with a very low d′ (Fig 9). A two-way ANOVA with factors neighboring channel and stereo (convex or concave) indicated a significant clustering of the 3D-structure preference in the large majority of selective channels (94% and 97% of stereo-selective neurons with a main effect of factor ‘neighboring channel’ in patients 1 and 2, respectively, and with 2 and 5 channels, respectively, showing an interaction between channel and stereo in these two patients). Since the high-gamma response correlates with population spiking activity [32,33], these results indicate clustering of the 3D-structure preference in human LOC, consistent with previous findings in monkey ITC [29].

## Discussion

We present the first report of intracortical recordings of SUA, MUA, and LFP activity in human LOC using microelectrode arrays. Our 96-electrode array with an interelectrode spacing of 0.4 mm allowed extensive neuronal recordings in human visual cortex with an unprecedented spatiotemporal resolution. Our experiments confirm the robust sensitivity of LOC neurons to image scrambling, as predicted by fMRI; reveal significant 2D-shape and 3D-structure selectivities at the level of SUA, MUA, and high-gamma responses; and provide the first RF maps of individual LOC neurons. Moreover, our data furnish new and crucial evidence concerning the microarchitecture of LOC, in that the shape preference differed drastically between neighboring electrodes spaced a mere 400 microns apart.

Our approach using a microelectrode array has several advantages compared to previous electrophysiological studies in humans. Subdural grids with contact points measuring several millimeters [3] sample neural activity over a wide area, whereas we used intracortical microelectrodes with sharp tips spaced just 400 microns apart, so that a single row of 10 electrodes occupied a stretch of cortex measuring only 3.6 mm. Furthermore, the microelectrode array allowed us to simultaneously record neural activity on 96 microelectrodes compared to recordings on only 2 microelectrodes in [11]. Finally, the spatial arrangement of the microelectrode array (10 by 10 electrodes) also allowed us to investigate the microarchitecture of human visual cortex at the scale of cortical columns. It should also be noted that our recording sites were located near the entry point of one of the depth electrodes and were, in retrospect, not part of the epileptogenic zone (EZ).

Ever since the original publication by Kourtzi and colleagues [17], numerous studies have used the LOC localizer [intact shapes–scrambled shapes] to identify shape-sensitive regions in human visual cortex. However, the actual underlying neural selectivity has never been revealed. An extensive body of work has employed visual adaptation, observed via fMRI activation, as an indirect measurement of neuronal shape selectivity in humans (e.g. [34]), but the interpretation of these effects and their relation to neural selectivity at the single-cell level remain controversial [35]. Here, we not only confirmed the strong effect of image scrambling on SUA, MUA, and high-gamma responses in LOC, but we also revealed significant response differences for intact shapes, i.e., shape selectivity, at the level of single neurons, as previously shown in the macaque ITC [36,37]. A bilateral lesion of LOC produces a profound deficit in shape recognition [38–40], similar to ITC lesions in monkeys [41–45], and transcranial magnetic stimulation over LOC also impairs shape discrimination [46,47]. Our data support the notion that these deficits arise from a loss of shape-selective neurons in LOC.

A substantial fraction of our MUA and LFP recording sites showed significant shape tuning—possibly as strong as in the monkey ITC [48]—indicating that shape preference may be localized within the LOC. In addition, the fixed arrangement of the microelectrodes, spaced 400 microns apart, allowed assessing the spatial organization of sensitivity to image scrambling and of the shape selectivity at a high spatial resolution. Our observation that the shape preference changed markedly over the extent of 400 microns is in line with previous studies in the macaque ITC demonstrating considerable clustering for shape features. Fujita and colleagues [20] showed that ITC neurons with similar shape selectivities are organized vertically across the cortical thickness, and Tsunoda and colleagues [49] used intrinsic optical imaging to highlight patches of activation elicited by specific object images spaced 0.4 to 0.8 mm apart. Thus, our results are highly consistent with previous studies in the ITC of macaque monkeys. Although the spatial scale of the clustering we observed (400 microns) was much smaller than the voxel size in most human fMRI studies (typically 2 × 2 × 2 mm), our findings corroborate the capacity of multivoxel pattern analysis (MVPA) to extract shape and depth information from fMRI data [50–52]. Similar observations have been made on orientation selectivity in early visual cortex of cats and humans [53]. Note also that MVPA cannot decode this information in the absence of clustering of the neural selectivity [54].

The spatially restricted nature of high-gamma responses we measured in LOC is consistent with previous studies in visual cortex indicating that the higher frequency bands of the LFP signal correspond with MUA [32,33,55,56] and originate from a small region of cortex measuring a few hundred microns in extent [57]. However, studies in auditory cortex reported considerable volume conduction—even in the high-gamma band of the LFP signal—over several millimeters of cortex [58]. Although our data do not allow us to fully resolve this controversy, it should be noted that our spatially selective recordings were obtained during an active fixation task, where no influence of anesthetics was possible, in contrast to the Kajikawa and Schroeder study. It should also be noted that although we recorded SUA in one patient, we were able to confirm our findings with MUA and high-gamma responses in both patients.

Our data also shed light on the RF properties of LOC neurons. Although we did not obtain sufficient data for an exhaustive RF description, a few conclusions seem to be warranted. The average RF size in LOC was large (473 degree^2^), and a considerable fraction of LOC neurons responded to stimuli presented in the ipsilateral hemifield. A previous study in human early visual cortex reported that the RF measured with high-gamma responses is even more restricted than that measured with MUA [11]. Thus, our observation that LOC sites frequently exhibit bilateral SUA, MUA, and high-gamma responses is interesting in view of the comparison between the human LOC and monkey ITC areas (see below). A limitation of our RF mapping resides in the fact that we used a relatively large stimulus (8.5 degrees), which might overestimate size and laterality of the RFs. The stimulus was also not adapted to the selectivity of the neural site. Future studies will have to investigate the RF profile of LOC neurons in more detail.

To our knowledge, we also provide the first evidence for 3D-structure selectivity defined by binocular disparity in human visual cortex. A number of recording sites showed differential responses to convex and concave surfaces (composed of the same monocular images), across different positions in depth, indicative of higher-order disparity or 3D-structure selectivity. Similar to the selectivity for image scrambling and that for individual shapes, the 3D-structure preference was highly localized on individual electrodes, since recording sites with strong selectivity were frequently located next to recording sites with a very low selectivity. A large number of studies [22,23,59–62] have investigated 3D-structure selectivity in the macaque anterior ITC (area TE). More recently, Verhoef and colleagues showed clustering of the MUA selectivity for 3D structure in area TE using identical stimuli [29], and microstimulation of these clusters could predictably alter the perceptual report of the animal in a 3D-structure categorization task. Hence, the 3D-structure selectivity we observe here has also been described in the macaque ITC. Moreover, patient DF [63], who suffered bilateral damage to LOC, was impaired in using the relative disparity between features at different locations, although 3D-structure categorization was not tested.

One major, outstanding question relates to the possible homologies between the different subparts of human LOC and monkey ITC areas TEO and TE [64]. Previous studies have linked the LO1 and LO2 subsectors to the monkey area TEO, since LO1 and LO2 subsector may be retinotopically organized [13,14], similar to TEO [15]. Although a single study cannot resolve this homology question, several observations we made are highly relevant in this respect. Shape-selective responses can be observed in TEO and in TE. However, the robust 3D-structure selectivity we observed was previously described in the more anterior part of area TE in macaques [22] but is virtually absent in macaque area TEO [65]. Moreover, the large and frequently bilateral RFs we observed in human LOC are more consistent with TE than with TEO [66]. Detailed mapping of RFs [15,67] and stimulus reduction during single-unit recordings [68] will clarify in greater detail the relationship between this part of the LOC and the monkey ITC areas.

Overall, the similarities between the human LOC and the (more anterior part of) monkey ITC were very apparent. In both species, neurons are sensitive to image scrambling [69] and are shape selective; shape preference is clustered; the RFs are large and include the fovea [70]; and neurons preserve their 3D structure preferences across position in depth [22]. The only possible discrepancy between our results in the human LOC and previous studies in monkey ITC may reside in the latencies of the neuronal response. When tested with images of objects, the MUA became selective only after 125 ms, and the response and selectivity latency for 3D stimuli equaled 125 and 225 ms, respectively. In contrast, macaque ITC neurons can signal shape differences starting at 70–80 ms after stimulus onset and at 80–100 ms for 3D stimuli [22]. However, the interpretation of these latency differences requires measurements at many different stages of the hierarchy in both species. Moreover, neural latencies might be influenced by anti-epileptic drugs, and nearly all invasive recording studies are performed in this population of patients. Our results are in line with previous single neuron studies in the human medial temporal lobe—which is downstream from the LOC—reporting latencies of 300 ms [5,8]. Previous LFP recordings using subdural grids/strips [71,72] along the human ventral visual stream showed latencies around 100–150 ms, consistent with the current study. However, neuronal latencies are highly influenced by the number of stimulus repetitions, therefore a higher number of selective recording sites and a higher number of trials may have yielded shorter latencies in our experiments. More detailed measurements in the two species in areas in which the homology is clearly established (e.g., V1) are undoubtedly necessary.

## Materials and methods

Ethical approval was obtained for micro-electrode recordings with the Utah array in patients with epilepsy (study number s53126). Study protocol s53126 was approved by the local ethical committee (Ethische commissie onderzoek UZ / KU Leuven) and was conducted in compliance with the principles of the Declaration of Helsinki (2008), the principles of good clinical practice (GCP), and in accordance with all applicable regulatory requirements. Strict adherence to all imposed safety measures, including case report forms (CRFs) and in detail reports on (serious) adverse events ([S]AEs), was required. All human data were encrypted and stored at the University Hospitals Leuven.

In patients with intractable epilepsy, invasive intracranial recording studies aimed to identify the EZ for subsequent removal; microelectrodes (Utah array) were additionally implanted to study the microscale dynamics of the epileptic network in the presurgical evaluation for research purposes. No additional incisions were made for the purpose of the study. The Utah array was placed on the convexity of the brain at the target location of—and in combination with—clinical electrodes, analogous to previous studies using micro-electrode arrays, such as [73–75]. Target locations for clinical electrodes were determined by the epileptologist and were based on preoperative electroclinical and advanced imaging investigations (including MRI, PET, SPECT/SISCOM). The location of the Utah array was always at the site of the presumed epileptogenic zone (PEZ) and away from eloquent brain areas, as determined by preoperative task-based motor and language fMRI. Since the PEZ dictates the location of array implantation, implants at the same site in different subjects occur only very rarely, which explains the low number of patients (*N* = 2).

In both patients (patient 1, 28-y-old female; patient 2, 44-y-old male), the PEZ covered the occipitotemporal cortex. During their surgery, microelectrode arrays were implanted in the right (patient 1) and left (patient 2) LOC, in an area of cortex with a high probability of resection provided that the presumed ictal onset zone was confirmed after invasive intracranial recordings. Patient 1 had undergone a surgical resection of a cystic lesion (WHO grade I ganglioglioma; MNI 28 to 46, −42 to −62, −5 to −27 mm) in the right occipitotemporal region 7 y earlier, with late seizure recurrence after initial seizure freedom. Patient 2 did not undergo a resection before invasive intracranial recordings, and in this MRI negative case ictal SPECT suggested a left posterior temporal onset. Both patients continued their anti-epileptic drugs during the study. Patient 1 was on brivaracetam 50 mg twice daily and perampanel 8 mg once daily; patient 2 was on valproate 500 mg twice daily and lamotrigine 200 mg twice daily. Intracranial recordings revealed an anterior temporal EZ in both patients, and they underwent a temporal lobectomy including the mesial temporal structures several months later.

Written informed consent in both patients was obtained before the start of the study. Possible additional risks of micro-electrode placement, such as superficial hemorrhage and infection were discussed on 2 different occasions (once at the outpatient clinic and again before surgery). The Utah array was used because this represents the only FDA-approved micro-electrode array, capable of studying the microscale dynamics of the epileptic network at the single-unit level, and because these arrays have been safely used in previous studies in both epilepsy patients [73–76] as well as in patients undergoing BMI implants [77–83]. Classical clinical electrodes have large contact surfaces and record from a large number of neurons (10^4^–10^5^) and thus are not capable of studying microscale dynamics. As these micro-electrode arrays are not CE marked, local regulatory approval (FAGG) was additionally obtained. Previous histological studies show that micro-electrode arrays cause focal tissue damage at the thin electrode trajectories upon insertion [84,85]. Postoperative MRI several weeks after electrode removal did not show any signal changes at the implantation site of both patients, indicating that there were no larger cortical lesions associated with array implantation.

To minimize trauma and risk of hemorrhage due to micro-electrode insertion, we performed under-insertion of the array with a low insertion pressure (15 psi). This implies that the pressurized inserter did not insert the electrodes over their full length, ensuring that the electrode pad on which the electrodes are bonded does not impact the brain. The risk of infection was minimized by using a custom percutaneous connector, with a small wire tunneled through the skin, in analogy to the classic clinical electrodes.

### fMRI

#### Stimuli

The stimuli were projected from a liquid crystal display projector (Barco Reality 6400i, 1,024 × 768 pixels, 60-Hz refresh rate) onto a translucent screen positioned in the bore of the magnet (57 cm distance). The patients viewed the stimuli through a mirror tilted at 45° and attached to the head coil.

#### LOC localizer

The LOC localizer stimuli were images measuring 300 × 300 pixels. We used grayscale images and line drawings of familiar objects (20 images and 20 line drawings), as well as scrambled versions of each set (Fig 1) [86]. The scrambled images were created by dividing the intact images into a 20 × 20 square grid and randomizing the positions of each of the resulting squares. The grid lines were present in both the intact and the scrambled images. The overall size of the stimuli measured 7° in visual angle, and each stimulus was presented for 1,000 ms.

#### Stereo localizer

The stimulus set consisted of random-dot stereograms in which the depth was defined by horizontal disparity (dot size 0.08°, dot density 50%, vertical extent 5.5°) and were presented on a gray background. All stimuli were generated using MATLAB (R2010a, MathWorks) and were gamma corrected. We used a 2 × 2 design with factors curvature (curved versus flat) and disparity (stereo versus control) (as described in [27,28,87,88]). The stereo-curved condition consisted of 3 types of smoothly curved depth profiles (1, 1/2, or 1/4 vertical sinusoidal cycle) together with their antiphase counterparts obtained by interchanging the right and left monocular images (disparity amplitude within the surface: 0.5°). Each of the 6 depth profiles was combined with one of 4 different circumference shapes and appeared at 2 different positions in depth (mean disparity + or − 0.5°), creating a set of 48 curved surfaces. In the stereo-flat condition, flat surfaces (using the same 4 circumference shapes) were presented at 12 different positions in depth, such that the disparity content (the sum of all disparities) was identical to that in the stereo-curved condition. Finally, the control conditions (stereo-control and flat-control) consisted of the presentation of one of the monocular images (either belonging to one of the stereo-curved stimuli or to one of the stereo-flat stimuli) to both eyes simultaneously. Each control condition consisted of exactly the same monocular images as the corresponding stereo condition, hence the binocular input was identical in the stereo conditions and in the control conditions. The overall size of the stimuli measured 5.6° in visual angle, and each stimulus was presented for 1,000 ms. Dichoptic presentation of the stimuli was achieved by means of red/green filter stereo glasses worn by the patient.

#### Data collection

Scanning was performed on a 3-T MR scanner (Achieva dstream, Philips Medical Systems, Best, the Netherlands) located at the University Hospitals Leuven. Functional images were acquired using gradient-echoplanar imaging with the following parameters: 52 horizontal slices (2 mm slice thickness; 0.2 mm gap; multiband acquisition), repetition time 2 s, time of echo 30 ms, flip angle 90°, 112 × 112 matrix with 2 × 2 mm in-plane resolution, and a sensitivity-enhancing (SENSE) reduction factor of 2. The 25 slices of a volume covered the entire brain from the cerebellum to the vertex. A 3D high-resolution (18181.2) T1-weighted image covering the entire brain was acquired in the beginning of the scanning session and used for anatomical reference (repetition time 4.6, time of echo 9.7 ms; inversion time 900 ms; slice thickness 1.2 mm; 256 × 256 matrix; 182 coronal slices; SENSE reduction factor 2.5). The single scanning session lasted 60 min.

#### CT

A CT scan (Siemens, 1 mm slice thickness, 120 kV, Dose length product of 819 mGy.cm) was performed 2 h after electrode placement to verify the location of the microelectrode array.

#### LOC localizer

Stimuli (shapes, line stimuli, scrambled shapes, scrambled line stimuli, fixation only, Fig 1) were presented in blocks of 24 s except for the fixation condition (20 s); each block was repeated 4 times in a run, creating runs of 464 s. Individual stimuli were presented for 1,000 ms (ISI = 0; fixation time: 200 ms).

#### Stereo localizer

Stimuli (curved stereo, flat stereo, curved control, flat control, fixation only) were presented in blocks of 24 s, and each block was repeated 4 times in a run, creating runs of 480 s. Individual stimuli were presented for 1,000 ms (interstimulus interval = 0 ms; fixation time = 200 ms). Twelve functional volumes were acquired for every block (or condition, each 24 s long), and these were embedded in a time series of 222 volumes (444 s).

#### Data analysis

Data analysis was performed using the SPM12 software package (Wellcome Department of Cognitive Neurology, London, UK) running under MATLAB (Mathworks, Natick, MA). The preprocessing steps involved (1) realignment of the images and (2) coregistration of the anatomical image and the mean functional image. Before further analysis, the functional data were smoothed with an isotropic Gaussian kernel of 5 mm. To determine the exact location of the Utah array, the CT scan was coregistered with the anatomical image using SPM12 software; 3D renderings of the brain were created using Freesurfer, on which the SPM volumes were warped.

#### LOC localizer

To localize areas responding more strongly to the presentation of objects versus scrambled controls, we calculated the contrast [shapes + outlines] − [scrambled shapes + scrambled outlines] at *p* < 0.05, FWE corrected.

#### Stereo

To identify regions sensitive to binocular disparity, we calculated the main effect of stereo: [curved stereo + flat stereo] − [curved control + flat control] at *p <* 0.05, FWE corrected.

### Electrophysiology

In 2 patients with intractable epilepsy, a 96-channel microelectrode array (4 × 4 mm; electrode spacing of 400 microns; patient 1 electrode length 1.5 mm, patient 2 electrode length 1 mm; Blackrock Microsystems, UT) was implanted to study the microscale dynamics of the epileptic network (study protocol s53126). The array was implanted according to the manufacturer’s protocol with a pressurized inserter wand.

#### Stimuli

All stimuli were presented by means of a custom-made stereoscope. Images from 2 LCD monitors were presented to the 2 eyes with the use of customized mirrors at a viewing distance of 56 cm (1 pixel = 0.028°). Continuous eye-movement tracking (left eye, 120 Hz; ISCAN, MA), ensuring fixation in an electronically defined window (3 × 3 degrees), was performed throughout the experiment. Trials in which the patients did not maintain fixation were aborted.

#### LOC localizer

The same stimulus set as in the fMRI experiment was used. The stimuli presented in the stereoscope were 8.5 degrees in size. After a brief period of fixation (200 ms), the stimulus was presented for 500 ms, followed by an interstimulus interval of 100 ms. In the LOC localizer, no disparity was present in the stimuli.

#### RF mapping

To map the RF, a single nonscrambled shape (8.5 degrees) was presented at 25 different positions in the visual field, covering 50 degrees horizontally and 30 degrees vertically, during passive fixation.

#### Stereo test

We presented concave and convex surfaces at 3 different positions in depth (near, at the fixation plane, and far; Fig 8A) at the fixation point while monitoring the position of the left eye. To avoid monocular depth cues, the disparity (disparity amplitude: 0.25 degrees) varied only along the surface of the shape, while the circumference of the shape was kept at a constant disparity (+0.25 degree, 0 degree, or −0.25 degree disparity) (as in [29,31]). The patients had to categorize the 3D structure of the stimulus (concave or convex, 100% disparity coherence) independently of the position in depth by means of a button press after stimulus offset (1,000 ms of stimulus presentation time) (as in [31]). An auditory tone provided feedback after every successfully completed trial. Both patients performed at more than 90% correct.

#### Data collection

Data were collected using a digital headstage (Blackrock Microsystems, UT) connected to a 128-channel neural signal processor (Blackrock Microsystems, UT). For LFP recordings, the signal was filtered with a digital low-pass filter of 125 Hz, and LFP signals were recorded continuously (sampling frequency: 1,000 Hz). Single- and multi-unit signals were high-pass filtered (750 Hz). A multi-unit detection trigger was set at a level of 95% of the signal’s noise. All spike sorting was performed offline (Offline Sorter 4, Plexon, TX).

#### Spike rate analysis

Data analysis was performed using custom-written Matlab (MathWorks, MA) software. For every channel, we calculated the net spike rate by subtracting the average baseline activity from the spike rate. Spike rate was further normalized by dividing the net spike rates by the average spike rate for the best condition (50–300 ms after stimulus onset) for each channel. Statistics were performed using permutation tests, where real data were randomly distributed over all the different conditions 1,000 times. The differences between 2 conditions were calculated for every permutation and compared with the actual difference between conditions. The latency of the spiking activity for visually responsive channels was defined as the first of 2 consecutive 50 ms bins with a spike rate higher than the average baseline plus 2 standard errors. The selectivity latency was defined as the first of 2 consecutive 50 ms bins with a spike rate for the preferred condition higher than the average spike rate for the nonpreferred condition plus 2 standard errors.

#### LFP analysis

For every trial, the time-frequency power spectrum was calculated using Morlet’s wavelet analysis techniques [89], with a spectrotemporal resolution equal to 7, after filtering with a 50 Hz notch filter (FieldTrip Toolbox, Donders Institute, the Netherlands [90]). This method provides a better compromise between time and frequency resolution compared to methods using Fourier transforms [91,92]. To remove any filter artifacts at the beginning and end of the trial, the first and last 100 ms of each trial were discarded. Power was normalized per trial by dividing the power trace per frequency by the average power for this frequency in the 100 ms interval before stimulus onset. To exclude trials containing possible artifacts in the LFP recordings, maximum values of the continuous LFP signal were determined, and trials with maximum values above the 95th percentile were removed. Furthermore, the data set was split in two, and all population analyses were repeated for both halves of the data independently, to check for consistency. We analyzed the LFP power in the high frequency bands (high-gamma): 80–120 Hz. Lowest frequencies had to be excluded from our analyses, as our trials were maximally 1 s long. All statistics on LFP data were obtained using permutation tests as described for spiking activity. The latency of the LFP response per frequency band was defined as the first of 5 consecutive timestamps (in milliseconds) in which the average power minus 2 standard errors was higher than 1 (= average power of the normalized baseline). The LFP latency for selectivity between conditions was defined as the first of 2 consecutive samples in which the average power for condition A minus 2 standard errors was higher than the average power for condition B.

*D-prime*: d′ statistics were calculated as follows:

$$d\prime =\left(\mu_{Pref}‑\mu_{NonPref}\right)/\sigma$$

Here, μ_Pref_ and μ_NonPref_ denote the mean responses to the preferred and the nonpreferred condition (e.g., nonscrambled versus scrambled), respectively, and

$$\sigma =\surd (\sigma 2_{Pref}+\sigma 2_{NonPref})/2$$

is the pooled variance of the two response distributions. This measure differs from those used in previous studies [32,93,94] in that it explicitly takes into account the trial-by-trial variability of the response [95].

To estimate the spatial extent of selectivity observed in the MUA and high-gamma band over the array, we determined for each visually responsive channel its immediate neighbors (i.e., either 8 channels, for recording channels that were not located on the edge of the array, or 5 channels for edge electrodes). We calculated a two-way ANOVA with factors scrambling (scrambled versus nonscrambled) and position for each channel individually. Significance was tested using *p* = 0.05.

#### Ranking

To investigate the MUA and high-gamma responses to individual stimuli, we applied a ranking technique in which individual nonscrambled stimuli were ranked based on the electrode’s average spiking activity and high-gamma power evoked by the stimuli, and then the same ranking was applied to the individual scrambled control stimuli. To investigate differences between rankings, a linear regression was performed, and a 95% confidence interval was used to determine significant differences between regression coefficients or intercepts. Finally, the same ranking technique was used to investigate the spatial specificity of the shape selectivity: we ranked the nonscrambled stimuli for each electrode based on the spiking activity and high-gamma responses, and this same ranking was then applied to the responses of all neighboring channels separately. We then averaged the spike rate and gamma responses for the ranked data of the neighboring channels to determine whether the shape preference was preserved at neighboring channels. Differences in ranking were investigated using a linear fit as described above.

#### RFs

The average SUA and high-gamma power were calculated during stimulus presentation for each stimulus position and filtered with a Gaussian (sigma: 0.5).

To calculate RF size, we constructed RF maps by interpolating the neuronal responses between all positions tested across the 50 × 30 degree display area and then calculating the number of pixels in the RF map with a response higher than 50% of the maximum response.

### Data deposition

Data are deposited in the Dryad repository: http://dx.doi:10.5061/dryad.qd4vd71 [96].

## Supporting information

S1 Fig Second example neuron. (A, C) This neuron responded more to intact shapes and lines than to their scrambled versions as shown in these spike raster plots. (B, D) Response to the 5 best and 5 worst stimuli, indicating that this neuron discriminated reliably between different shapes. scr, scrambled.
(TIF)

S2 Fig Ranking of lines for MUA and high-gamma LFP for both patients. The same ranking is applied for the neighboring channels and the corresponding scrambled control stimuli. An overview of the array is shown as inset, each square represents an electrode, illustrating that electrodes with high d′ values (response nonscrambled versus scrambled) are neighbored by electrodes with low d′ values. The underlying data can be found at 10.5061/dryad.qd4vd71. LFP, local field potential; MUA, mulit-unit activity.
(TIF)

S3 Fig Average high-gamma of 3D-structure–selective sites for each patient. Preferred versus nonpreferred shapes. The underlying data can be found at doi:10.5061/dryad.qd4vd71.
(TIF)

S4 Fig Overlap of fMRI activations. LOC localizer activation (contrast [shapes + outlines] − [scrambled shapes + scrambled outlines]). Stereo localizer activation (contrast [curved stereo + flat stereo] − [curved control + flat control]). *p* < 0.05, FWE-corrected for multiple comparisons. The underlying data can be found at doi:10.5061/dryad.qd4vd71. fMRI, functional magnetic resonance imaging; FWE,; LOC, lateral occipital complex.
(TIF)

## Abbreviations

3D: three-dimensional
CT: computed tomography
CRF: case report form
EEG: Electroencephalography
EZ: epileptogenic zone
fMRI: functional magnetic resonance imaging
FWE: family-wise error
GCP: good clinical practice
hMT: human middle temporal visual area
ITC: inferior temporal cortex
LH: left hemisphere
LFP: local field potential
LO1: subsector 1 of lateral occipital complex
LO2: subsector 2 of lateral occipital complex
LOC: lateral occipital complex
MNI: Montreal Neurological Institute
MUA: multi-unit activity
MVPA: multi-voxel pattern analysis
PEZ: presumed epileptogenic zone
RF: receptive field
RH: right hemisphere
SAE: serious adverse event
SENSE: sensitivity-enhancing
SUA: single-unit activity
(area) TE: anterior part of inferotemporal cortex
TEO: posterior part of inferotemporal cortex
