# Correction: Single-cell selectivity and functional architecture of human lateral occipital complex

**DOI:** 10.1371/journal.pbio.3000588

**Published:** 2019-12-06

**Authors:** Thomas Decramer, Elsie Premereur, Mats Uytterhoeven, Wim Van Paesschen, Johannes van Loon, Peter Janssen, Tom Theys

In Table 1, the values for the left hemisphere (LH) hMT x-value and the right hemisphere (RH) hMT x-value are incorrect. The LH hMT x-value was previously listed as 54 and should be -54. The RH hMT x-value was previously listed as 21 but should be 51.

**Table 1:** MNI coordinates. Position of the micro-electrode array in relation to the centroid of fMRI activations in the literature [18,19]. Coordinates are shown in MNI space for the LH and RH.

	LH			RH		
	x	y	z	x	y	z
Patient 1				55	−71	1
Patient 2	−55	−77	6			
LOC ant	−46	−54	−11	29	−49	−16
LOC post	−59	−82	6	31	−82	−9
LO1	−31	−93	−4	31	−92	−3
LO2	−38	−85	−6	37	−84	−6
hMT	−54	−74	14	51	−67	3

The corrected Table 1 is provided below, the legend remains unchanged.
