# Supplementary figures and images for: Single-cell selectivity and functional architecture of human lateral occipital complex
Source: PLoS Biol. 2019 Sep 12;17(9):e3000280. doi: 10.1371/journal.pbio.3000280 (PMC6759181; doi:10.1371/journal.pbio.3000280)

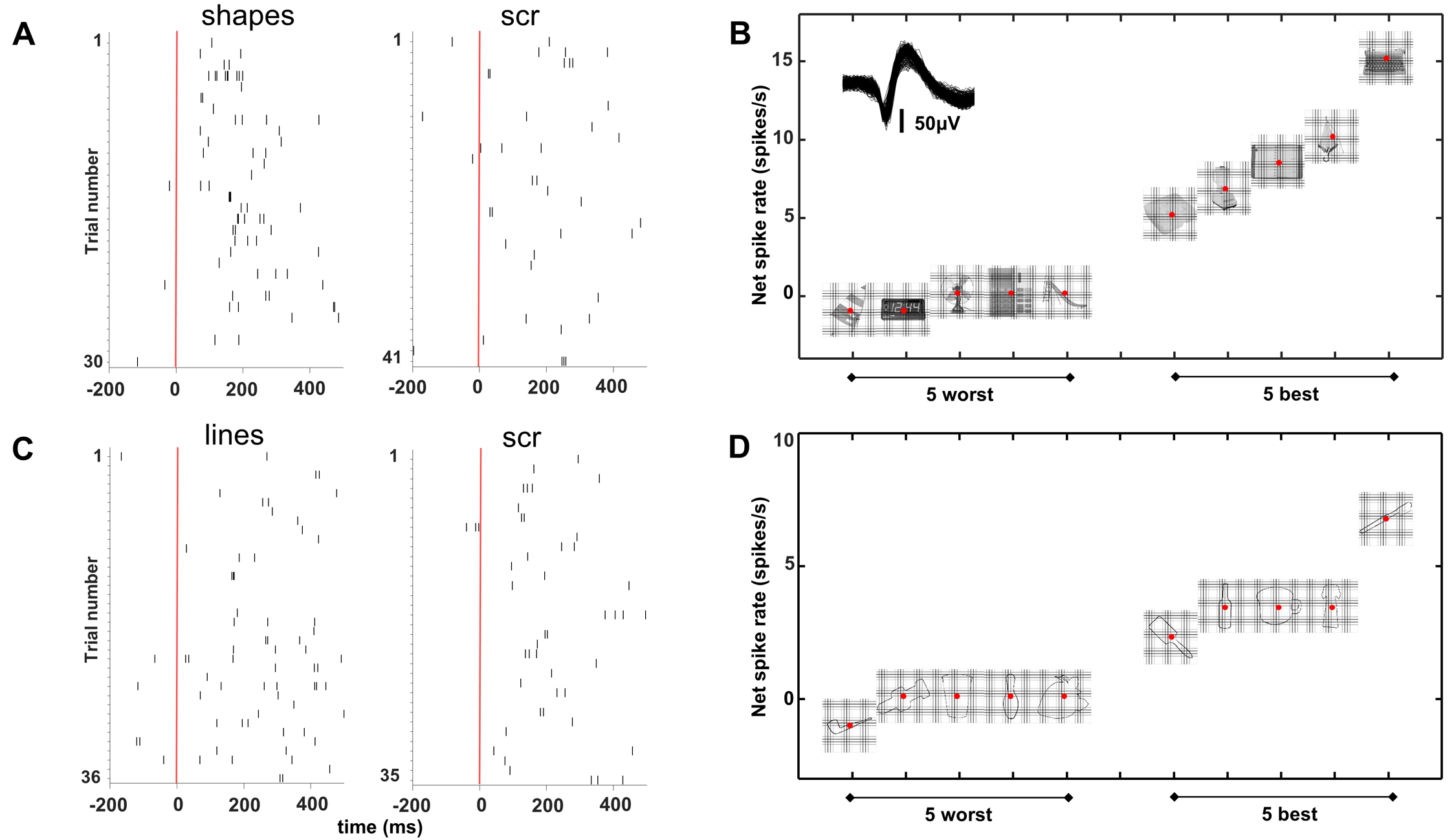

Supplement: S1 Fig — (A, C) This neuron responded more to intact shapes and lines than to their scrambled versions as shown in these spike raster plots. (B, D) Response to the 5 best and 5 worst stimuli, indicating that this neuron discriminated reliably between different shapes. scr, scrambled. (TIF) [file pbio.3000280.s001.tif]

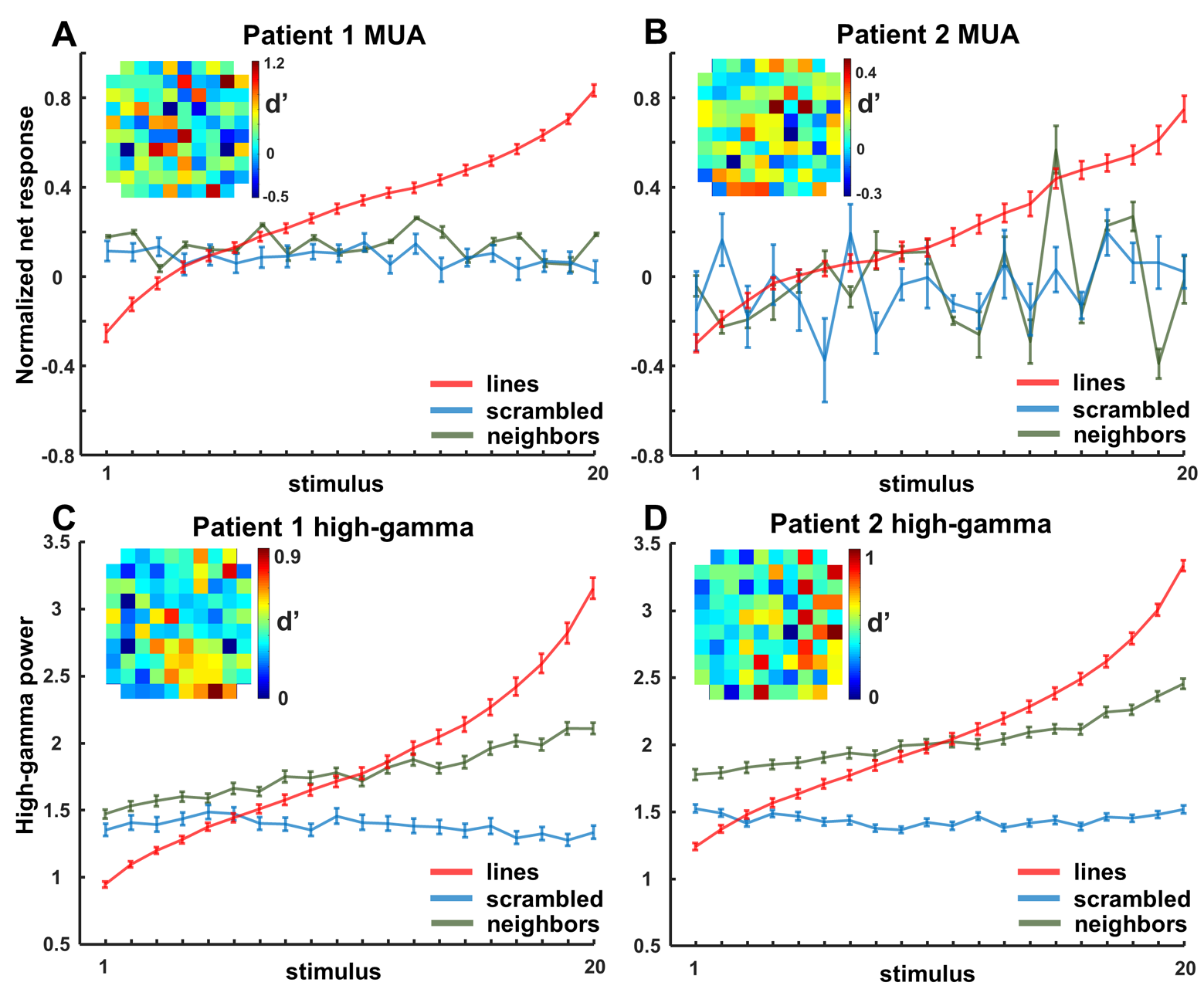

Supplement: S2 Fig — The same ranking is applied for the neighboring channels and the corresponding scrambled control stimuli. An overview of the array is shown as inset, each square represents an electrode, illustrating that electrodes with high d′ values (response nonscrambled versus scrambled) are neighbored by electrodes with low d′ values. The underlying data can be found at 10.5061/dryad.qd4vd71. LFP, local field potential; MUA, mulit-unit activity. (TIF) [file pbio.3000280.s002.tif]

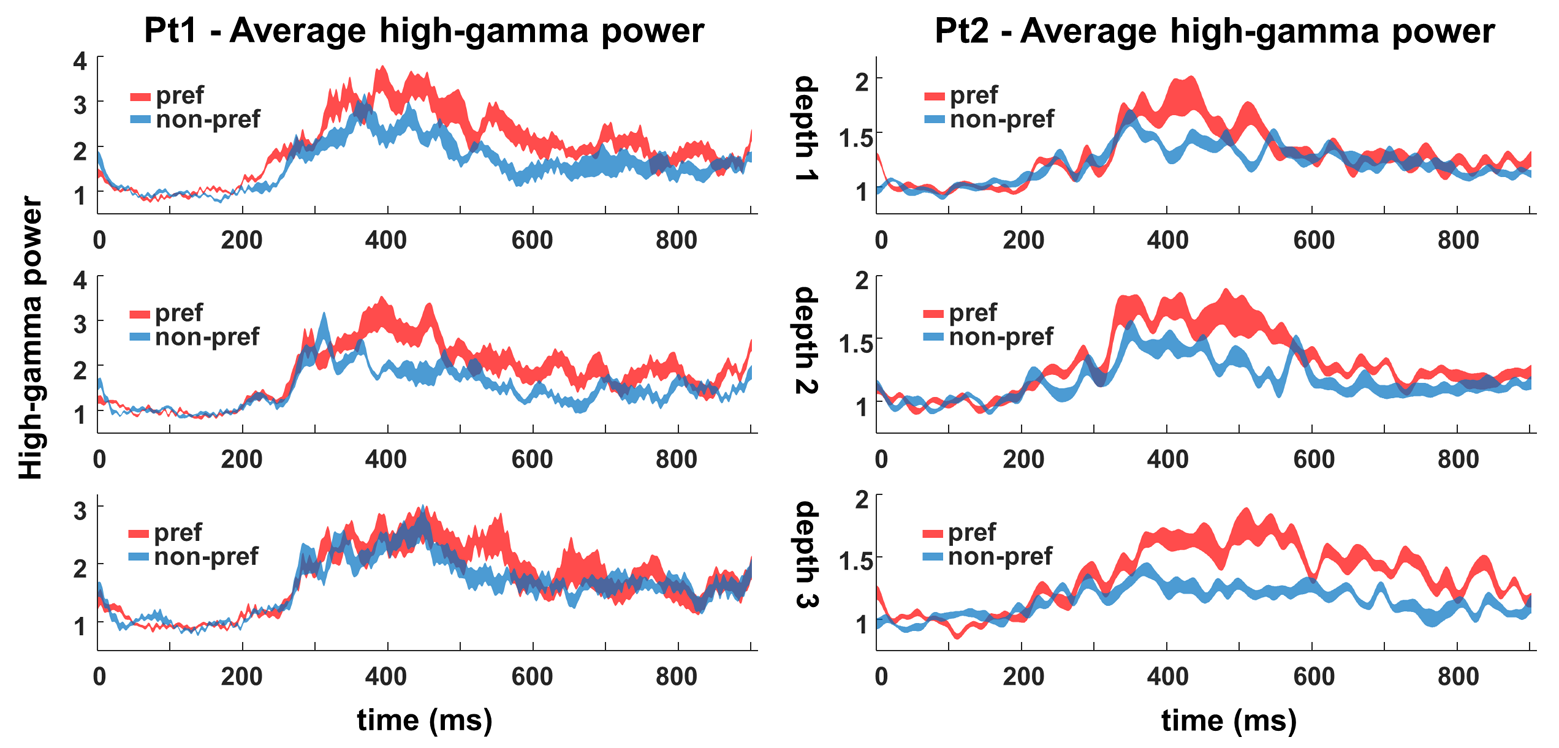

Supplement: S3 Fig — Preferred versus nonpreferred shapes. The underlying data can be found at doi:10.5061/dryad.qd4vd71. (TIF) [file pbio.3000280.s003.tif]

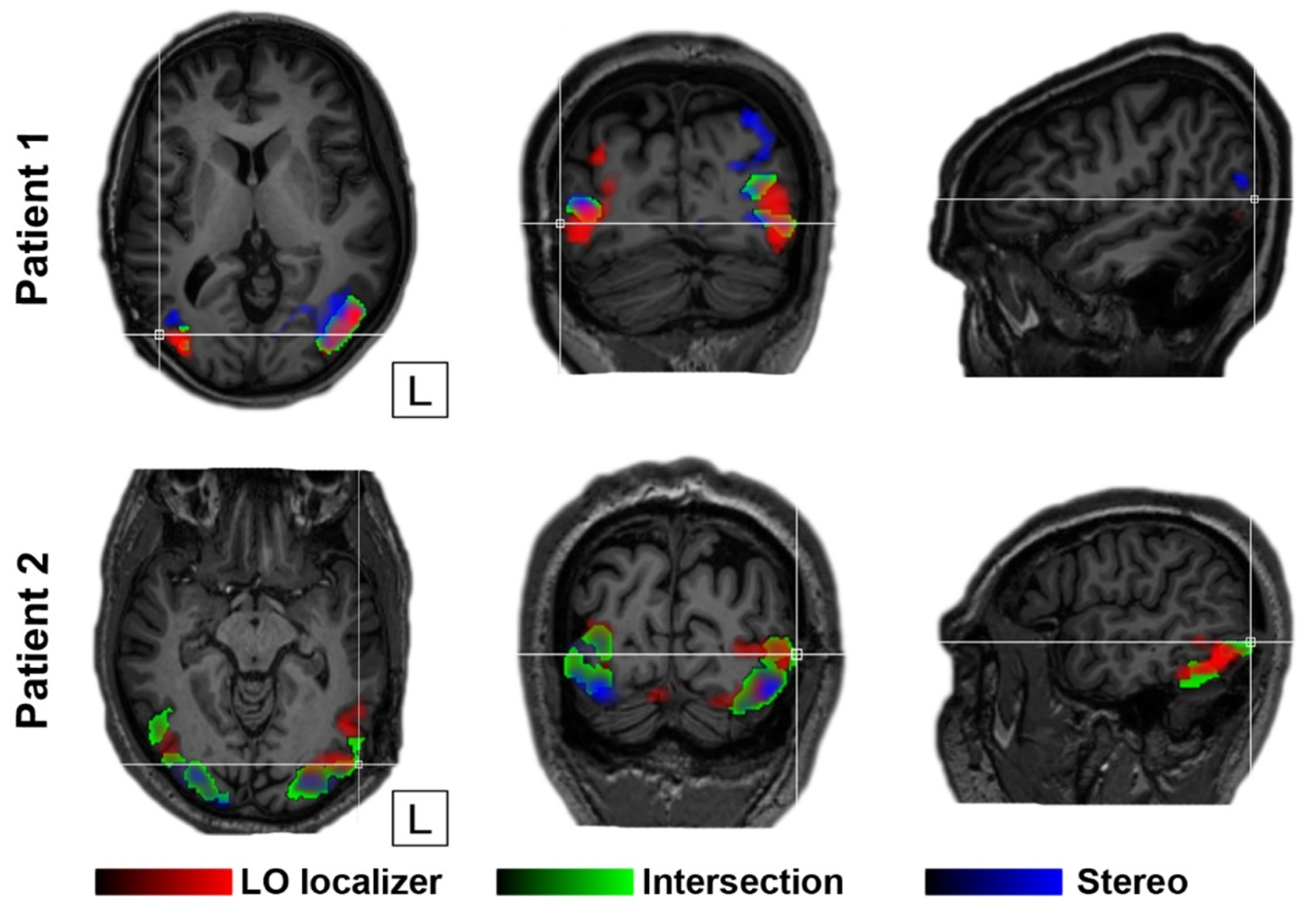

Supplement: S4 Fig — LOC localizer activation (contrast [shapes + outlines] − [scrambled shapes + scrambled outlines]). Stereo localizer activation (contrast [curved stereo + flat stereo] − [curved control + flat control]). p < 0.05, FWE-corrected for multiple comparisons. The underlying data can be found at doi:10.5061/dryad.qd4vd71. fMRI, functional magnetic resonance imaging; FWE,; LOC, lateral occipital complex. (TIF) [file pbio.3000280.s004.tif]
